# Liability for Maliciously Filing Information Charging Insanity

**Published:** 1897-08

**Authors:** 


### LIABILITY FOR MALICIOUSLY FILING INFORMATION CHARGING INSANITY.

The Supreme Court of Iowa affirms in the case of Comfort v. Young, Jan. 22, 1897, a judgment of $750 for the plaintiff, who sued to recover damages for an alleged libelous publication that consisted of an information, filed by the defendant with the board of insane commissioners, charging that the plaintiff was insane, and a fit subject for custody and treatment in the insane hospital of the State. The defense was made that the information was a privileged communication. Persons have the undoubted right, says the Supreme Court, to file such informations as the one in question, when made in good faith, and in the honest belief that the statements therein made are true. But one can not use such instrumentalities for the express purpose of gratifying his malice or indulging his passions without making himself answerable to the law. It is for the trial judge to determine whether the publication complained of was privileged or not, and, if privileged, whether absolute or conditional; but it is for the jury to find whether the proceeding was instituted through malice.—*Journal of the American Medical Association.*

